# Correction to *Lancet Glob Health* 2017; 5: e593–603

**DOI:** 10.1016/S2214-109X(17)30307-8

**Published:** 2017-08-11

*Habib MA, Soofi S, Cousens S, et al. Community engagement and integrated health and polio immunisation campaigns in conflict-affected areas of Pakistan: a cluster randomised controlled trial.* Lancet Glob Health *2017; **5:** e593–603*—In this Article, included children were stated to be younger than 24 months of age in the summary and the legend of table 6, this has been corrected to 60 months. In the summary and figure 2, the number of households visited and surveyed, children assessed, and clusters in the trial profile has been corrected. In table 1, in arm C 10566 has been corrected to 10556. In tables 3, 4, and 5, data have been corrected for rounding errors and the corresponding data in the text has also been corrected. In table 3, the p value 0·21 has been corrected to 0·2139. In the fourth paragraph of the Results, (6·9–10·1) has been corrected to (6·8–10·1), and in the seventh paragraph (82·6–88·6) has been corrected to (82·6–88·8). There were also rounding errors in the appendix, which have also been corrected. These corrections have been made as of Aug 11, 2017.

